# Optimizing Population Variability to Maximize Benefit

**DOI:** 10.1371/journal.pone.0143475

**Published:** 2015-12-09

**Authors:** Leighton T. Izu, Tamás Bányász, Ye Chen-Izu

**Affiliations:** 1 Department of Pharmacology, University of California, Davis, Davis, California, United States of America; 2 Department of Physiology, University of Debrecen, Debrecen, Hungary; 3 Department of Medicine, University of California, Davis, Davis, California, United States of America; 4 Department of Biomedical Engineering, University of California, Davis, Davis, California, United States of America; São Paulo State University, BRAZIL

## Abstract

Variability is inherent in any population, regardless whether the population comprises humans, plants, biological cells, or manufactured parts. Is the variability beneficial, detrimental, or inconsequential? This question is of fundamental importance in manufacturing, agriculture, and bioengineering. This question has no simple categorical answer because research shows that variability in a population can have both beneficial and detrimental effects. Here we ask whether there is a certain level of variability that can maximize benefit to the population as a whole. We answer this question by using a model composed of a population of individuals who independently make binary decisions; individuals vary in making a yes or no decision, and the aggregated effect of these decisions on the population is quantified by a benefit function (e.g. accuracy of the measurement using binary rulers, aggregate income of a town of farmers). Here we show that an optimal variance exists for maximizing the population benefit function; this optimal variance quantifies what is often called the “right mix” of individuals in a population.

## Introduction

Variability is inherent in all populations. In manufacturing, variability has been called“the enemy of mass production” [1] and decades of research and effort have gone into reducing variability between parts [2, 3]. On the other hand, variability between individuals is the *sine qua non* of biological evolution. Whether variability in a population is beneficial, detrimental, or inconsequential is not often clear. Agriculture provides some classic examples. The potato blight epidemic of 1845–1852 that caused massive starvation in Ireland [4] occurred in part because of overdependence on a narrow range of species that happened to be susceptible to disease. Yet the detrimental effects of narrow diversity are often outweighed by the benefits of product uniformity and high productivity. Intuitively, it is plausible that there might be an optimal level of variability or diversity among individuals that could maximize the total population benefit in both productivity and adaptability.

Here, we analyze how the variance between individuals can affect some measure of benefit to the population as a whole. The model we use is a population composed of heterogeneous individuals that make binary decisions; individuals vary in making a yes or no decision, and the aggregate effect of these decisions on the population is quantified by a benefit function. Such a model can be used to describe various populations, such as a town of farmers with each individual deciding on whether or not to plant tulips that year, or a population of binary measuring devices with distributed thresholds. The benefit function is defined in a way to quantify some form of benefit to the population. The benefit function for the farmer example is the aggregate income of all farmers in town; for the measuring devices, it is the accuracy of measurement. Here we show that an optimal variance exists that maximizes the population benefit function.

## Methods and Analysis

### Mathematical framework

To make the quantitative link between variability in a population and population benefit we need to (a) model how individuals in a population behave and (b) quantify the effects of the individuals’ behavior. We use a simple behavioral model where an individual makes a binary decision based on whether a signal, called *L*, exceeds a threshold. Variability is introduced by assuming that the threshold differs between individuals. The probability distribution of individual thresholds, or simply the *threshold distribution*, is *ϕ*(*L*, *s*), where *s* is the measure of variability.

The effect of all the individual’s behavior is measured by the *population benefit function* *B*(*L*, *s*), which depends on both the signal level (*L*) and the variability (*s*).

The threshold distribution *ϕ*(*L*, *s*) and the population benefit function *B*(*L*, *s*) constitute our mathematical framework to study how variability affects population benefit. The problem we will solve is this: *For a given signal L and threshold distribution ϕ*(*L*, *s*), *what level of variability s maximizes the population benefit B*(*L*, *s*)?

### Sloppy Rulers

To answer this question, we consider the problem of measuring the length of an object with a population of binary rulers. We start with this example for three reasons. First, in this case the meaning of *ϕ*(*L*, *s*) and *B*(*L*, *s*) are easily understood. Second, this measurement problem requires us to develop a method, called the Sloppy Algorithm, that finds the optimal value of variability *s* that maximizes the benefit function *B*(*L*, *s*) for any *L*. Third, there is a surprising equivalence between making accurate measurements with binary rulers and maximizing income in a town of farmers.

The problem is to measure an object of length *L* where 0 ≤ *L* ≤ 1 using a population of *N* binary rulers of unit length. These binary rulers have only a single mark engraved *approximately* about the midpoint. Because the position of the mark varies from ruler to ruler they are called “sloppy rulers.” While making measurements with such rulers might seem contrived and unrealistic, this process, in fact, always occurs when determining the least significant digit of any measuring device. For example, in an 8-bit analog-to-digital converter (ADC) with a 5 volt full-scale range, the quantal step size is 5V/256 = 19.5 mV, which we can take as our unit length. *L* is now some voltage between 0 and 19.5 mV scaled between 0 and 1. The last bit will be set to 0 or 1 depending on whether *L* is less than or greater 0.5. In an ADC the scaled voltage at which the bit switches between 0 and 1 (in this case 0.5) is called the threshold voltage. By analogy, we now call the graduation mark on a sloppy ruler the threshold.

It is important that the threshold of each of the *N* rulers be distributed approximately about the midpoint. If the mark on all *N* rulers were exactly at 1/2 (as in an ADC) and *L* happened to be 0.4 then all we can say is that the object has length slightly less than 1/2. But as we will show, if the thresholds are randomly distributed in “proper” way then *L* can be accurately determined with any degree of resolution. The proper way will be the distribution with optimal variability.

The length of an object using the sloppy rulers is estimated as follows: Align the left edges of the object and ruler. Each ruler *i* casts its “vote” of *λ*_*i*_ = 0 (vote no) or *λ*_*i*_ = 1 (vote yes) depending on whether the right edge of the object is below or above the threshold at *ρ*_*i*_, respectively. The *threshold distribution* *ϕ* is the distribution of threshold locations, *ρ*_*i*_. We initially assume *ϕ* is the normal distribution with mean *μ* = 1/2 and standard deviation SD = *s*. The estimate of *L*, $\bar{\lambda }$, equals the number of yes votes, *n*, multiplied by 1 (length of the ruler), divided by the total number of votes *N*, $\bar{\lambda }=\left(n\times 1\right)/N$. We allow the position of the graduation mark to range from −∞ to ∞ (possibly running off the ruler). Because the ruler length is 1, $\bar{\lambda }$ numerically equals the probability that *L* is greater than *ρ*, *P*(*L* > *ρ*), which as *N* → ∞, is

$$\begin{array}{ccc} \lim_{N\to \infty }\bar{\lambda }=P\left(L>\rho \right) & = & \int_{-\infty }^{L}\phi \left(z;\mu =1/2,s\right)\;dz=\frac{1}{2}\left[1+\text{erf}\;\left(\frac{\sqrt{2}\left(2L-1\right)}{4s}\right)\right]\\ & \equiv & Q(L,s) \end{array}$$ (1)

This equation gives the relationship between the estimate $\bar{\lambda }$ and the variability *s*.

We now define a benefit function. The benefit function reflects the worth or merit we ascribe to $\bar{\lambda }$ and so may be defined in many ways. When measuring length, a natural definition of benefit would indicate how close $\bar{\lambda }$ is to the true length *L*. One such population benefit function, *B*, is given by

$$B\left(L,s\right)=1-\frac{\left|Q(L,s)-L\right|}{\epsilon_{\max}}=1-2\left|Q\left(L,s\right)-L\right|.$$ (2)

*ϵ*_max_ is defined as the supremum (least upper bound) of *L*_1_ (the 1-norm) errors

$$\epsilon_{\max}=\sup\left\{\left|Q(L,s)-L\right|,L\in \left[0,1\right],\;s\geq 0\right\}.$$ (3)

From the definition of *Q* it follows that *ϵ*_max_ = 1/2 so *B*(*L*, *s*) ranges from 0 to 1. The population of rulers that has the *optimal level of variability*, s*, estimates the length exactly. Perfect accuracy and maximal benefit are achieved when s* solves the fixed point problem

$$Q(L,s^{*})=L.$$ (4)

### The Sloppy Algorithm

At first glance there appears to be a logical problem of needing to know *L* in the first place in order to get s*. However, we escape this dilemma by proving that for any *L*, s* can be found iteratively and that the estimate $\bar{\lambda }$ converges monotonically to *L* using the following “Sloppy Algorithm”:

1. Choose *s*_0_ arbitrarily.
2. Compute ${\bar{\lambda }}_{1}=Q\left(L,s_{0}\right)$.
3. For any *i* > 1,
  $$\;\text{choose}\;s_{i}\;\text{such}\;\text{that}\;Q\left(\lambda_{i},s_{i}\right)=\lambda_{i}.$$ (5)
  The solution to Eq (5) is
  $$s_{i}\left(\lambda_{i}\right)=\frac{\sqrt{2}\left(2\lambda_{i}-1\right)}{4{\text{erf}}^{-1}\;\left(2\lambda_{i}-1\right)}.$$ (6)
4. The next value *λ*_*i*+1_ is computed from
  $${\bar{\lambda }}_{i+1}=Q\left(L,s_{i}\right)$$ (7)

Steps [3] and [4] are the recursion rules and are repeated as many times as desired.

**Fig 1:**
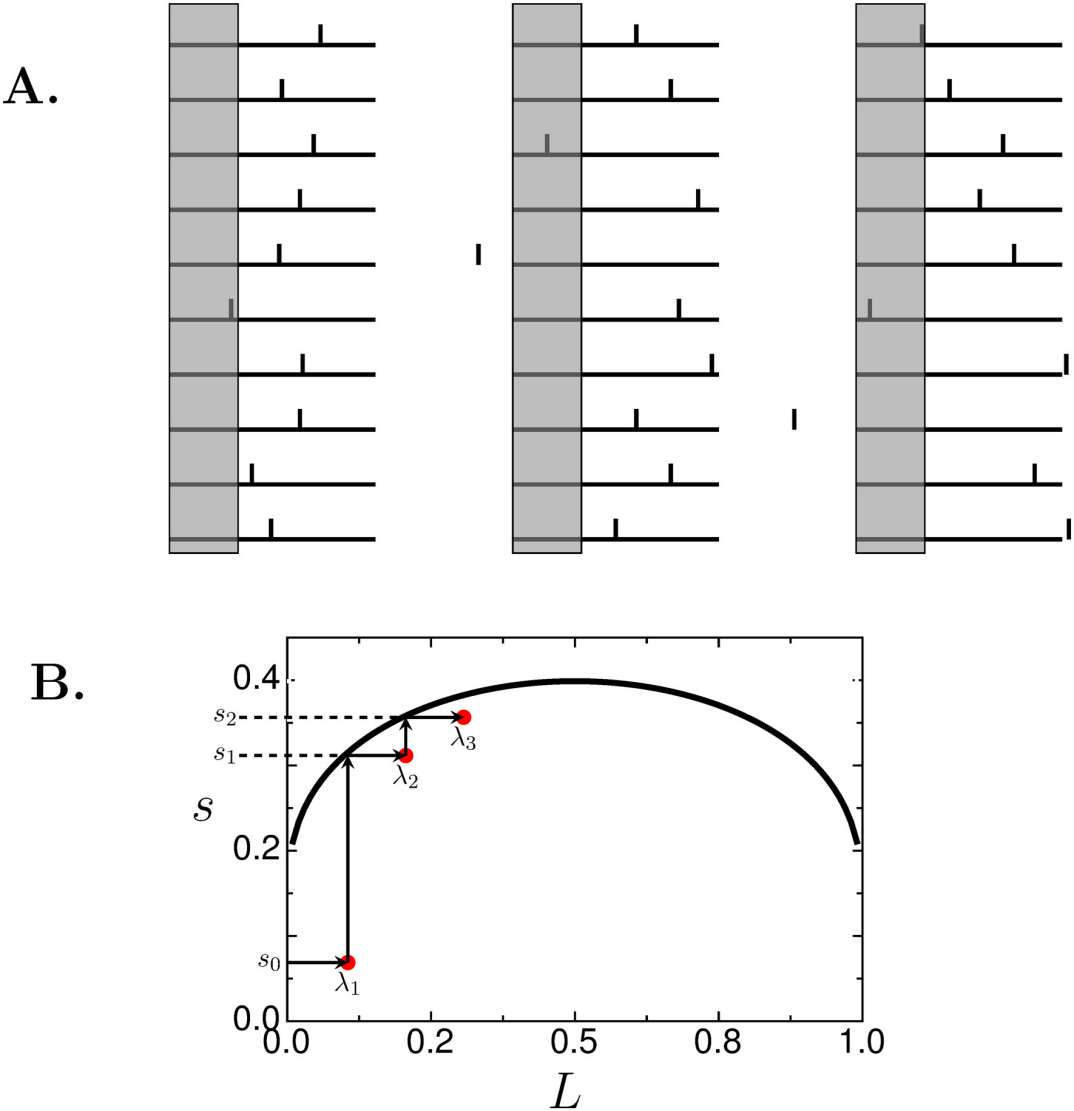
Schematic of recursion rules of the Sloppy Algorithm. (**A**), Width of gray bar is to be measured with rulers of unit length (horizontal lines). Vertical tick lines are the graduation marks. Note that a graduation mark can lie beyond the edge of the ruler as seen in the middle and rightmost set of rulers. (**B**), *s*, solves the fixed point problem *Q*(*L*, *s*) = *L*. Arrows show how *s*_*i*_ and *λ*_*i*+1_, generated by this ruler example, are linked to each other and how the algorithm converges.

Fig 1 illustrates how the Sloppy Algorithm works. The leftmost drawing in Fig 1A shows 10 rulers with thresholds (short vertical lines) normally distributed about the center with standard deviation of *s*_0_ = 0.1. For this first step the value of *s*_0_ can be any value >0. For *step 2* we compute *λ*_1_ by counting the number of times the right edge of the object (gray bar), whose length is *L* = 0.3, lies to the left of all the thresholds. In this case *n* = 1 so *λ*_1_ = 0.1. For *step 3*, we compute *s*_1_ by solving *Q*(*λ*_1_, *s*_1_) = *λ*_1_, using Eq (6) or reading off the graph of *s*(*L*) in Fig 1B, which gives *s*_1_ = 0.312. For *step 4* a new set of rulers is created with thresholds drawn from a normal distribution with SD *s*_1_ (middle set of rulers) and we get the new estimate *λ*_2_. In this case, the right edge of the object lies above the threshold of two rulers so *n* = 2 and *λ*_2_ = 0.2. Repeating *step 3* we get *s*_2_ = 0.356 based on *λ*_2_ = 0.2. Repeating *step 4* (rightmost set of rulers) gives *n* = 3 so *λ*_3_ = 0.3, which happens to be the correct length. Steps 3 and 4 can be repeated as many times as desired. Fig 1B shows how *s*_*i*_ and *λ*_*i*+1_ are linked stepwise and the convergence of the algorithm.

**Fig 2:**
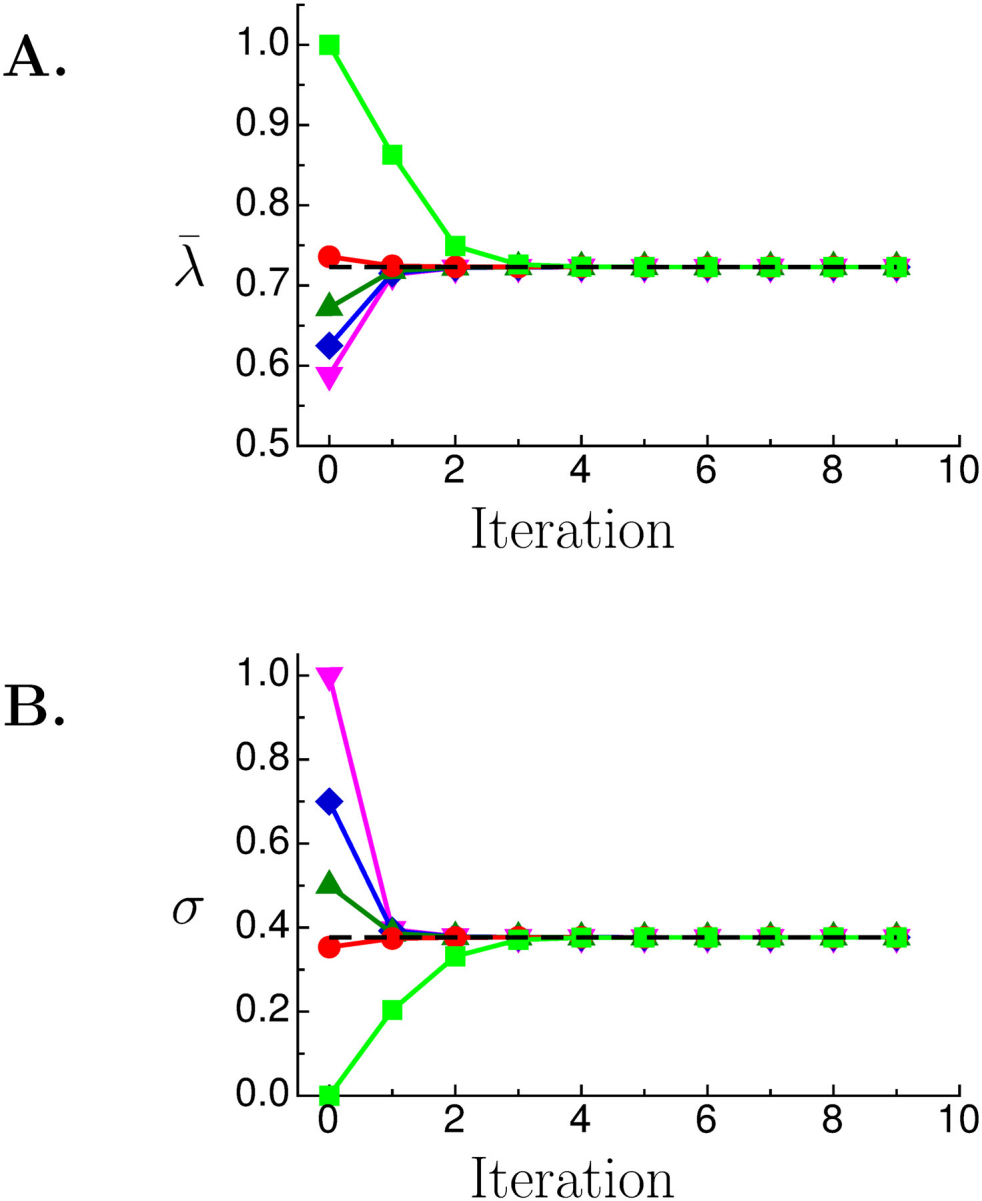
Convergence of the Sloppy Algorithm. Convergence of ${\bar{\lambda }}_{i}$ (**A**) and *s*_*i*_ (**B**) when the Sloppy Algorithm was used with different initial values of *s*; *s*_0_ = 10^−5^, light green, square; magic number, red, circle; 0.5, green, up-triangle; 0.7, blue, diamond; 1.0, magenta, down-triangle. Dashed black line marks the correct values of *L* = 0.723 and s* = 0.377.

Fig 2A and 2B show the convergence of ${\bar{\lambda }}_{i}$ and *s*_*i*_ for 5 different initial values of *s*_0_ that range from 10^−5^ to 1. In this case the true value of *L* is 0.723 (chosen arbitrarily) so s*(0.723) = 0.377; their values are shown by the black dashed lines. Notice that regardless of the initial value of *s*_0_, within 2 or 3 iterations ${\bar{\lambda }}_{i}$ already converges to within a few percent of *L*.

It can be shown that an ensemble of measuring devices is mathematically equivalent to a single device making multiple measurements. To illustrate this, we show, in Section 4 in S1 File, that a person, embedded in the Sloppy Algorithm iteration loop, can determine the absolute gray scale value of an image. The convergence to the correct gray scale value, shown in **Fig E** in S1 File, is similar to Fig 2.

The Sloppy Algorithm is not limited to *ϕ* being normally distributed and the right hand side of Eq (4) can be replaced by any strictly monotonically increasing function *f*(*L*) bounded by 0 and 1. We prove that the Sloppy Algorithm converges everywhere monotonically when *f*(*L*) = *L* and *ϕ* is the normal distribution in Section 1 in S1 File. Fig 2A and 2B show *λ*_*i*_ and *s*_*i*_ converging monotonically to the correct solution.

### Using the Sloppy Algorithm to achieve high-resolution measurements from low-resolution instruments

It may be surprising that a population of sloppy rulers can make accurate, high resolution measurements even though each ruler, having just a single mark, is of the lowest possible resolution. Sloppy rulers, when combined with the Sloppy Algorithm, provide a useful method to obtain accurate, high-resolution measurements. This method differs from dithering [5, 6], which is also used to increase measurement resolution. Section 5 in S1 File explains how they differ mathematically and the different arenas where they are useful.

There are two costs in using sloppy rulers. First, high resolution measurements require many rulers. To get three-digit resolution requires *N* ≥ 1000 rulers. Second, accuracy requires using the correct *s*, which the Sloppy Algorithm finds iteratively. As Fig 2 shows, it can take from one to three iterations to get an accurate estimate of *L*.

**Fig 3:**
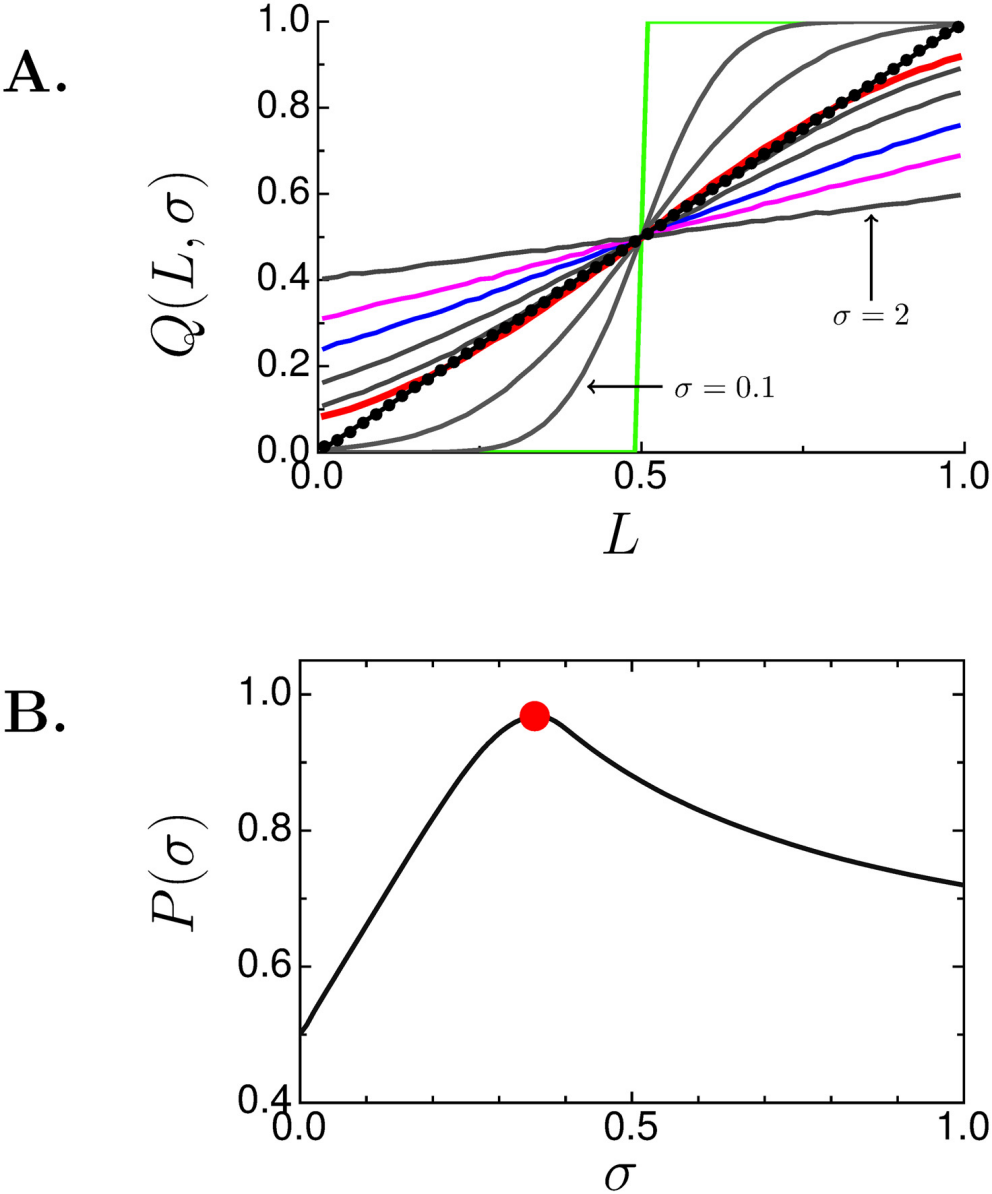
Sloppy rulers without Sloppy Algorithm. (**A**), length estimate (*Q*(*L*, *s*)) when *s* is fixed to 0 (light green), 0.1, 0.2, magic number (red), 0.4, 0.5, 0.7 (blue), 1 (magenta), and 2. Perfect fit falls on the diagonal line (circles). (**B**), performance *P*(*s*) given by Eq (8). Maximum occurs at the magic number $\sqrt{2}/4\approx 0.35$ (red circle).

Fig 3A shows what happens if the wrong value of *s* is used. The *x*-axis is the true length *L* and the *y*-axis is the estimate of *L* given by *Q*(*L*, *s*). Every *L* requires a specific s*(*L*) that gives the correct estimate *Q*(*L*, s*) = *L* and this s* is found using the Sloppy Algorithm. The black circles are these estimates and they fall exactly on the line *L* = *L*. Each solid curve shows the estimate made with a fixed value of *s*; the estimates are all wrong except at *L* = 1/2 and at most two other values of *L*. The red curve, however, merits special attention because it shows a “short cut” to find reasonably accurate solutions without the cost of iterating.

### The “magic number distribution”

Although no *single* value of *s* produces an exact estimate for all values of *L*, we see that the red curve in Fig 3A is close to the unit slope line except near 0 and 1. This curve was generated using the standard deviation *s*_*m*_ called the “magic number.” We derive the value of the magic number, $s_{m}=\sqrt{2}/4\approx 0.35$ in Section 2 in S1 File. We call the distribution *ϕ*(*L*, *s*_*m*_) the *magic number distribution*.

The magic number distribution is important because this single distribution could replace the infinite number of distributions (indexed by s*(*L*)) needed to get *perfect* benefit. To assess how well the magic number distribution can do this we need to gauge how close a distribution gets to being perfect for all *L*. This gauge is called the *performance*, *P*(*s*), defined as the average of *B*(*L*, *s*) over all values of *L*

$$P\left(s\right)=1-\frac{\int_{0}^{1}\;\left|Q\left(L,s\right)-L\right|\;dL}{\int_{0}^{1}\;\epsilon_{\max}\;dL}=1-2\int_{0}^{1}\;\left|Q\left(L,s\right)-L\right|\;dL.$$ (8)

Like *B*(*L*, *s*), *P*(*s*) ranges between 0 (worst performance) and 1 (perfect performance).

*P*(*s*), shown in Fig 3B, is maximized at the magic number (red circle). The performance at the magic number is 97% of the maximum value of 1. Perfect performance is impossible with a single distribution. However, the magic number distribution is the *optimal single distribution* that best approximates the infinitude of exact solutions obtained by the Sloppy Algorithm. This means that a population of sloppy rulers drawn from the magic number distribution can make good estimates of *L* with high resolution (given enough rulers) *without* iterations. This is of practical importance as it might be very difficult to manipulate the underlying variability of the threshold distribution even once, much less iteratively. Manipulating the threshold distribution is easy with rulers or voltage comparators (used in ADCs) but when the individuals are people manipulating thresholds might be difficult though possible [7].

Because the magic number distribution gives a good estimate for almost all *L*, starting the Sloppy Algorithm loop using *s*_*m*_ as the initial guess produces an initial estimate *λ*_0_ that will be almost always close to the correct value. This is shown by the red traces in Fig 2 where *s*_*m*_ was used as the starting guess.

### Optimizing variability for maximum benefit: An economic example

Measurements with sloppy rulers might not appear to have any bearing on how diversity of crops or people affect population benefit but we will show there is a very close relationship between making accurate measurements and maximizing aggregate income of the population.

To better understand how variability affects population benefit and performance, imagine a town of farmers who independently choose to plant tulips or the less valuable beans. One might initially predict that every farmer working for his/her own best interest would plant tulips. However, when faced with the uncertainty that the tulip market might be glutted, some farmers might choose to grow beans given its certain marketability. Let us assume that a farmer overcomes his/her concern for a tulip glut and plants tulips when the demand for tulips exceeds his/her risk threshold. Some farmers are risk-takers, others are risk-averse. Assuming there is a distribution of risk threshold in the farmer population, we ask what is the optimal level of threshold variability that maximizes the aggregate income of all the farmers. The aggregate income might not be important to the individual farmer but is important to the population, that is, the town. The larger the aggregate income, the more the town flourishes.

Define the benefit $\tilde{B}$, as the revenue collected by a town based on the following assumptions: (a) the income to the town increases at a rate of *b*_1_ per tulip up to the market demand *D* but receives no income for the tulips in excess of the demand. (b) Farmers not planting tulips plant beans whose value per farmer, *b*_2_, is less than that of tulips. Planting tulips incurs an *opportunity cost* by forgoing other activities such as planting beans. (c) A penalty, *b*_3_, is assessed per tulip for wasted resources in producing a number of tulips that is above or below the demand.

Suppose each farmer can produce *β* tulips; the town of *N* farmers can fulfill a maximum demand *D*_*m*_ = *Nβ*. The number of tulip farmers needed to exactly meet the demand *D* is n* = *D*/*β*. Define *L* to be the ratio of demand to maximum demand, *L* = *D*/*D*_*m*_ (0 ≤ *L* ≤ 1), so *D* = *βNL*. The normalized benefit *B* is defined as the ratio of benefit $\tilde{B}$ to maximum demand

$$\begin{array}{ccc} B\left(L,\nu \right)\equiv \frac{\tilde{B}\left(L,\nu \right)}{D_{m}} & = & \left[H(\nu )\nu -H(\nu -1)(\nu -1)\right]Lb_{1}\\ & + & \frac{b_{2}}{\beta }\left(1-L\nu \right)-L\left|1-\nu \right|b_{3} \end{array}$$ (9)

**Fig 4:**
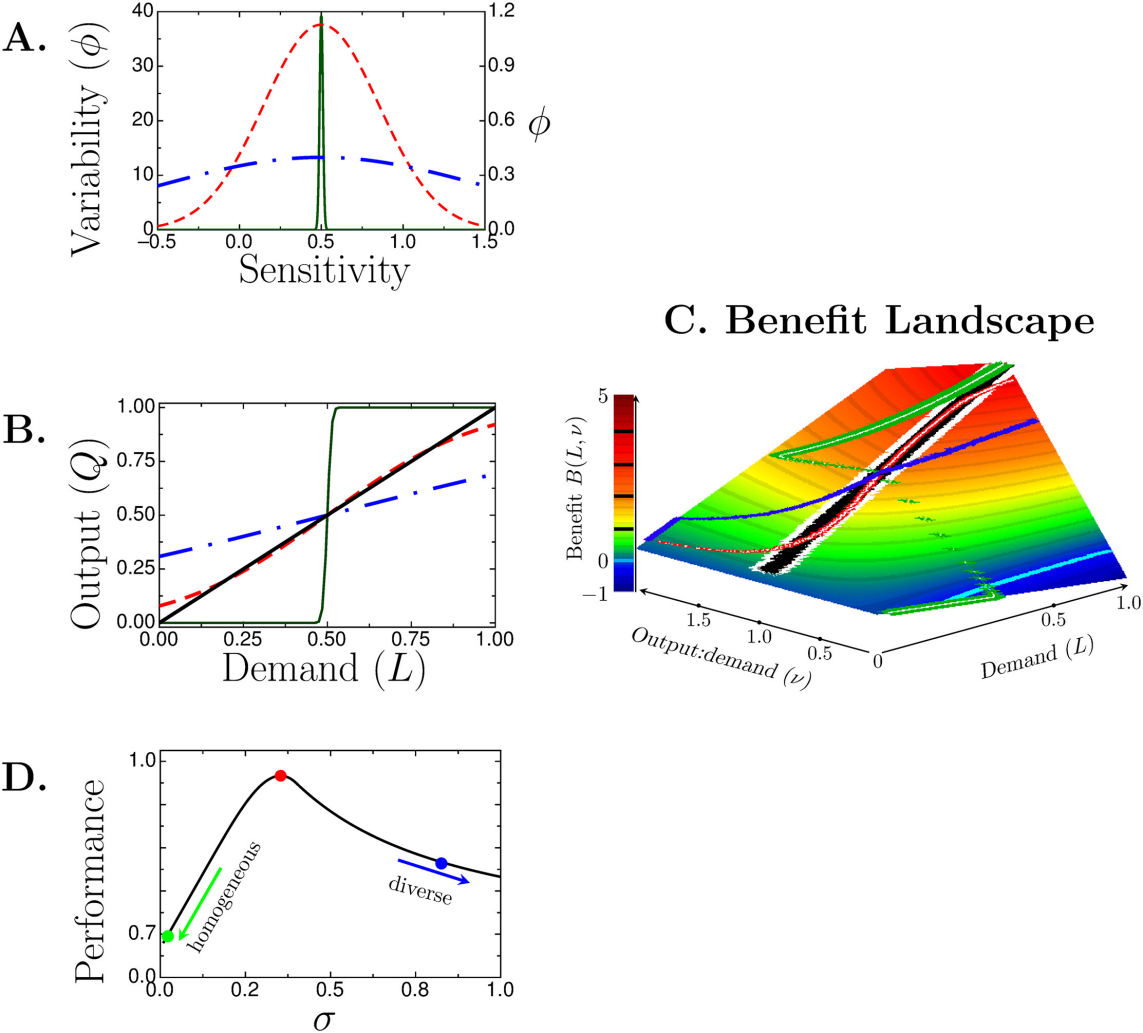
Farmer example. (**A**) Probability density function (*ϕ*) of farmers’ sensitivities. *s* = 0.01, green (solid, left axis); magic number, red (dashed, right axis); 0.8, blue (dash-dot, right axis). (**B**) Farmers output relative to demand. Solid black line shows perfect matching between output and demand. Curves’ colors and line patterns match those in A. (**C**), benefit landscape, *B*(*L*, *ν*). Thick white line lies on the ridge where *B* is maximized. Cyan curve near the *L*-axis is where *B* = 0; *B* < 0 for points below the curve (closer to *L*-axis) and *B* > 0 above the curve. Other colored curves are the benefits derived from farmer tulip output shown in panel (**B**). Black, Sloppy Algorithm; red, *s* = magic number; green, 0.01; and blue, 0.8. (**D**) Performance as a function of population variability *s*. Circles mark *s* = 0.01 (green), magic number (red), and 0.8 (blue).

*H* is the Heaviside function. *ν* is the ratio of the number of farmers planting tulips, *n*, to the number needed to meet the demand, *ν* = *n*/n*. We get an important interpretation of *ν* by rewriting it as *ν* = *NQ*(*L*, *s*)*β*/(*LNβ*), which is the *output to demand ratio*. The first term on the right hand side of Eq (9) is the income derived from tulips; it increases linearly until *ν* = 1 then flattens because the town receives nothing by producing more tulips than demanded. In the second term, 1 − *Lν* is the fraction of farmers planting beans. We assume that the farmers earn more by planting tulips than beans so *βb*_1_ > *b*_2_. The third term is the penalty for over- or under-producing tulips. Note that *B*(*L*, *ν*) is maximized when *ν* = 1, when tulip output exactly matches demand. Fig 4C shows the *benefit landscape* *B*(*L*, *ν*). The thick white line at *ν* = 1 demarcates the ridge of maximal benefit.

To link the benefit function to the farmer threshold distribution, suppose that each farmer independently decides whether to plant tulips or beans. The farmers’ decisions are based on their risk threshold, which we assume is normally distributed about the mean of *D*_*m*_/2 with standard deviation *s* ⋅ *D*_*m*_. Eager farmers start planting as soon as there is any demand while sluggards hold off until the demand becomes high. The fraction of farmers whose sensitivity is less than *L* is *Q*(*L*, *μ* = 1/2, *s*). The number of tulip planters is then *n* = *NQ*(*L*, *s*) (where *μ* = 1/2 is understood) so *ν* = *NQ*(*L*, *s*)/*NL*, which equals the ratio of the town’s tulip output to the demand. Because *B*(*L*, *ν*) is maximized when *ν* = 1, when output equals demand, it follows that the optimal variability s* solves *Q*(*L*, s*) = *L*. This fixed point problem is identical to that of the ensemble of sloppy rulers (Eq (4)). This means that the problem of getting accurate measurements with sloppy rulers is identical to the problem of efficient labor allocation in this simple economy.

Just as a population of sloppy rulers whose graduation marks have optimal s* variability can estimate the value of *L* exactly, a population of farmers whose sensitivities have variability s* will produce exactly the amount of tulips as the market demands. For every demand *L* there is a unique optimal variability in the farmers’ sensitivity s*(*L*) that results in a perfect matching of output to demand. If the threshold distribution can be altered with appropriate rewards or punishments [7], it is possible to use the Sloppy Algorithm to adjust *s* so that the collective output of the town’s farmers exactly matches the demand. However, this may be neither practical nor necessary. As with the sloppy rulers, if the farmers’ threshold distribution is the magic number distribution then their collective tulip output would almost exactly match the demand and thereby nearly maximize the benefit to the town. Importantly, this matching of output to demand occurs spontaneously by individual farmers making independent decisions, without needing a manager to dictate how many farmers in the town should plant tulips.

### The benefits of optimal variability

To see how variability amongst farmers affects the collective benefits, consider three towns having farmers with different risk threshold distributions shown in Fig 4A. The farmers’ output (*Q*, black line) relative to the demand (*L*) is shown in Fig 4B. In the town with a homogeneous population (green curves in Fig 4A and 4B), the farmers work in lock step and produce no tulips when the demand is low and too much when the demand is high. The benefit to the town is shown by the green trace on the benefit landscape in Fig 4C. Although this town produces no tulips when the demand is very low (*L* < 0.2) the town derives some positive benefit by planting beans (indexed by *b*_2_). For more moderate demand (0.2 < *L* < 0.5) the penalty for not producing tulips (the *b*_3_ term) outstrips the income from beans and the town suffers a deficit (*B* < 0, green curve dips below *B* = 0 indicated by the cyan-colored curve). Once the demand exceeds 0.5, all the farmers in this town plant tulips. The benefit increases but overproduction occurs (Fig 4B). The net benefit is submaximal because overproduction is penalized and, because all farmers are planting tulips, the town forfeits income from beans.

In contrast, the town of farmers with an overly broad distribution of risk thresholds (Fig 4A, blue dot-dashed curves) produces too much when the demand is low and too little when the demand is high (Fig 4B). The benefit this town derives is submaximal (blue curve, Fig 4C) because it it penalized for overproduction at low demand and also penalized for underproduction of valuable tulips because too many farmers plant the less valuable beans.

The town of farmers with the magic number distribution (red, dashed curve, panel Fig 4A) outputs tulips in near perfect accord with demand (Fig 4B) and the benefit (red-white curve in Fig 4C) is close to the theoretical perfect line except when the demand is very low because then too many farmers plant tulips.

To quantify how the town’s benefit changes with *s*, we define the performance *P*(*s*) as the ratio of the integral of the benefit (Eq (9)) over all *L* for a given *s* to the maximal achievable benefit,

$$P\left(s\right)=\frac{\int_{0}^{1}\;B\left(L,\nu (s)\right)\;dL}{\int_{0}^{1}\;B\left(L,\nu (s^{*}\left(L\right))\right)\;dL}.$$ (10)

*P*(*s*) ranges from 0 (zero income) to 1 (maximal achievable income). *P*(*s*) is shown in Fig 4D. The town with the magic number distribution (red circle) performs the best with *P*(*s*_*m*_) = 98%. By contrast, the homogeneous town (*s* = 0.01, green circle) has a performance 67%, and the overly diverse town (*s* = 0.8, blue circle) has a performance of 83%.

### Optimal variability depends on the population benefit function

We can intuitively understand how a group of people who function well to accomplish one goal might perform poorly when given another goal. Here we quantify how performance changes when the benefit function is redefined. The key result of this section is that the optimal level of variability depends on the benefit function.

Let us define a new benefit function

$$B\left(L,\nu \right)=\frac{f(L)}{L}\nu -\frac{\nu^{2}}{2},$$ (11)

where *L* and *ν* have their same meaning as in the farming example. Differentiating *B* with respect to *ν* shows that *B* is maximized along *ν* = *f*(*L*)/*L*. This implies that the population output is optimal when the variability s* solves *Q*(*L*, s*) = *f*(*L*). For both the ruler and farming cases, *f*(*L*) equaled *L*. Now suppose that *f*(*L*) is the cubic function *f*(*L*) = *L* − (*γ*/2)*L* ⋅ (*L* − 1/2) ⋅ (*L* − 1) for 0 ≤ *γ* ≤ 4 (these constraints keep *f*(*L*)≥0). s* is found for any *L* using the Sloppy Algorithm.

**Fig 5:**
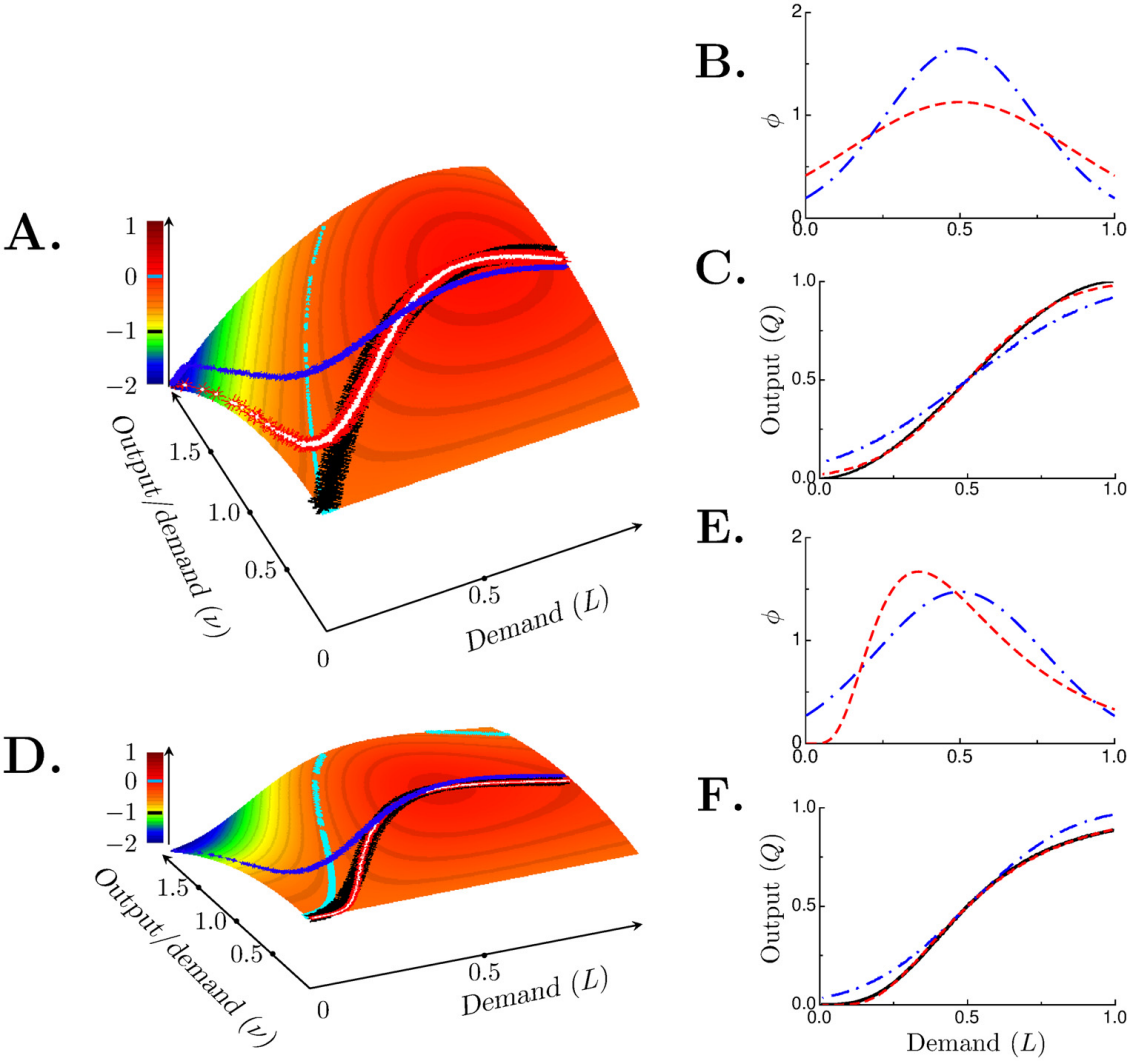
Benefit function determines optimal distribution. (**A**), benefit landscape defined by Eq (11) when *f*(*L*) is cubic (*γ* = 4). (**B**), normal distributions with *s* = 0.242 (red, dashed curve) or 0.354 (blue, dot-dashed curve). The former is the magic number for this benefit function while the latter is the magic number of the benefit function in the tulip farmer case. (**C**) shows the corresponding outputs of these populations; black curve is *f*(*L*). Sloppy Algorithm output matches *f*(*L*) exactly. Heavy black curve in panel (**A**) lies along the ridge of maximal benefit. Red (with white dots added for clarity) and blue curves on the benefit landscape are the benefit corresponding to the outputs in panel (**B**). (**D**), benefit landscape defined by Eq (11) when *f*(*L*) is sigmoidal. (**E**), magic number lognormal (red, dashed curve) and magic number normal (blue, dot-dashed curve) population decision models. The magic numbers are *s*_*m*_ = 0.559 (shape factor) for the lognormal and *s*_*m*_ = 0.271 (standard deviation) for the normal distribution. (**F**), outputs from corresponding distributions; black curve is *f*(*L*). Heavy black curve in panel (**D**) mark the ridge of maximal benefit. Red (with white dots) curve is the benefit for the lognormal distribution and the blue curve is for the normal distribution. Cyan-colored curves in (**A**) and (**D**) show where *B*(*L*, *ν*) = 0; points closer to the demand axis are positive.

The benefit landscape is shown in Fig 5A. The black curve shows the ridge where *B* is maximal. To achieve maximum benefit s* must be used, that is, for every demand *L* a different population must be used. As with the farmers, population diversity might not be practically changeable. Therefore we would like to see if there is a single distribution that produces near maximal benefit for all *L*. In other words, is there a magic number distribution?

In the case where *f*(*L*) = *L* and *ϕ* is normal, we could determine the magic number *s*_*m*_ analytically. In other cases, such as the one considered now, *s*_*m*_ is defined as the value that maximizes the performance *P*(*s*) (Eq (10)) and is found numerically.

Here, it turns out that *s*_*m*_ = 0.242 and *ϕ*(*L*, *s* = 0.242) is shown in by the red trace in Fig 5B. The output *Q*(*L*, *s*_*m*_) (red trace in panel C) for this magic number distribution matches very closely the ideal output *Q*(*L*, s*) (black trace). The benefit, shown by the red-white curve in Fig 5A, deviates significantly from maximal only when the demand is low. The performance of this magic number distribution is 0.94.

Suppose the town of farmers with the magic number distribution ($s_{m}=\sqrt{2}/4\approx 0.35$) that fared well with the benefit function defined by Eq (9) is now evaluated with the new benefit function (Eq (11)). How would this town fare? This town’s distribution and output are shown by the blue traces in Fig 5B and 5C. The benefit (blue curve in Fig 5A) deviates markedly from maximal. This town’s performance was 0.98 for the earlier benefit function (Eq (9)), but for the new benefit function, its performance is a paltry 0.037. For this new benefit function, this town is too diverse (*s*_*m*_ = 0.35). A modest reduction in the variability to *s*_*m*_ = 0.242 (making the town’s farmers slightly more homogeneous) will boost the town’s performance 25-fold to 0.94.

This example shows that the optimal level of variability in a population depends strongly on the benefit function. A population that is optimally diverse for one benefit function might do very poorly when the definition of benefit changes.

### Optimal variability depends on the threshold distribution

In this section we show that performance depends not only on the benefit function but also on the type of underlying threshold distribution. Up to now we assumed that the threshold distribution was normal. However, the lognormal distribution occurs frequently in biology [8, 9]. For example, mRNA [10] and protein levels [11, 12] are distributed lognormally. Furthermore, the magnitude of a cellular response is often a sigmoidal function of the signal amplitude. We therefore use the benefit function given above (Eq (11)) where *f*(*L*) is now the sigmoidal function *L* ^*n*^/(0.5^*n*^ + *L* ^*n*^). We compare how two threshold distributions, lognormal and normal, fare with this benefit function. The Sloppy Algorithm will find the optimal shape factor and the standard deviation, for the lognormal and normal distribution respectively, that maximizes the benefit function. When these optimal values are used the output is given by the black trace in Fig 5F and the benefit is maximal as shown by the black curve in Fig 5D.

As before we would like to replace the continuum of threshold distributions that give perfect benefit (*B*(*L*, s*) = 1) with a single distribution that gives good performance. Magic numbers were calculated for both models. For the lognormal distribution, the magic number for the shape factor is *s*_*m*_ = 0.559; for the normal distribution, the magic number is *s*_*m*_ = 0.242. The red trace in Fig 5E is the lognormal magic number distribution and the blue trace is the normal magic number distribution. The output for the lognormal distribution (red trace in Fig 5F) is very close to the optimal (black trace) while the output of the normal distribution (blue trace) deviates considerably. The corresponding benefits are shown by the red-white and blue curves in Fig 5C. Consonant with near perfect output, the performance of the population with the lognormal distribution is 0.999. The normal distribution population’s performance is only 0.727.

What accounts for the differences in performances of these two distributions? The lognormal distribution works well because the function *Q*(*L*, *s*_*m*_) using the lognormal “looks like” the sigmoidal *f*(*L*) while *Q*(*L*, *s*_*m*_) using the normal distribution does not “look like” *f*(*L*). By “looks like” we mean close in the *L*_∞_ norm (see Section 3 in S1 File).

This example shows that for a given benefit function, there might be no single population distribution of a particular type (normal in this example) that performs well. However, another type of population distribution (lognormal here) might work very well. Whether it is feasible to shape the population decision model will depend, of course, on the nature of the population.

## Discussion

### Reframing the diversity debate

What is the impact of variability or diversity in a population? This question has profound implications for industry, agriculture, bioengineering, and also for social institutions. Reasonable and compelling arguments have been made to support the beneficial effects of either diversity or homogeneity in a population. For example, some have called for supporting a diverse population of computer operating systems to thwart the widespread infection by computer viruses and worms [13, 14]. Others have countered that having a few operating systems allows programmers to concentrate their efforts to reduce vulnerabilities to cyber-attacks in these few [15]. Similarly, agriculture and forestry reap the benefits of product uniformity, high yields, and ease of management by using a small range of species. But catastrophic failure can occur should these few species succumb to disease or pests [4, 16–18]. An economy based on a broader diversity of crops is unlikely to fail catastrophically but might not enjoy the benefits of high productivity. The question on the value of diversity in society has sparked intense and often contentious social and political debates. The landmark Supreme Court decision of Regents of the University of California v. Bakke (438 U.S. 265 (1978)) hinged on the principle that a diverse student body is essential to the quality of higher education. But as with computers and agriculture, either broad or narrow diversity may bring trade-offs. A study by Putnam showed that broadly diverse communities had lower trust amongst its citizens than less diverse ones [19], but greater creativity had also been seen in more diverse groups [20, 21]. Another study by Watson et al. [22] showed that in the workplace, heterogeneous groups initially had more difficulty working together but after seventeen weeks they surpassed the homogeneous group at problem identification and generating alternative solutions.

Is it better to have diversity or homogeneity? The often contentious debate about the value of diversity in the workplace, schools, and society often stems, in part, from the Procrustean attempt to force the effects of diversity into a good-or-bad dichotomy. We argue that this is the wrong question. Instead, the proper and useful question is “What level of variability maximizes population benefit?”

To our knowledge only a few studies have addressed this question. Sih and Watters [23] measured the mating activity in a population of water striders as a function of the proportion of inactive, or moderately aggressive, or hyper-aggressive males in a group. They found that mating activity (a type of benefit) was a hump-shape function of the aggressiveness level, which is qualitatively similar to the performance curves we show in Figs 3B and 4D. Groups dominated by inactive males did not mate frequently and groups dominated by hyper-aggressive males drove females away.

### New insights on the existence and persistence of variability

In biological systems variability is ubiquitous and sometimes puzzling. Phenotypic variations occur even in genetic clones. Johannsen showed that genetically identical bean plants produced beans with broadly distributed sizes [24]. Recent studies extend Johannsen’s bean size variation to findings of broad variations in protein levels between isogenic cells [10–12] and in behaviors such as latency to enter S-phase [25] and apoptosis time [11]. Phenotypic variability is puzzling because in a static and spatially homogeneous environment, natural selection is supposed to favor the most fit, eliminate the rest, thus *narrowing* the range of variability [26–28]. However, the world is neither static nor homogeneous over long time. In a fluctuating and unpredictable environment a species that maintains a broad set of phenotypes, a strategy known as “bet-hedging”, might have a better chance of survival over multiple generations [28–31]. Nevertheless, could phenotypic variability be important on short time scales? Our work now provides a new insight into why variability is important to the population even in the lifetime of an individual.

The key to understanding this is to shift the focus from individuals to the population. This shift of focus allows us to see so called “cheaters” in a new light. Yeast cannot directly metabolize sucrose but when grown in a sucrose medium they express invertase, which cleaves sucrose into metabolizable glucose [32, 33]. Because invertase is expressed on the cell membrane, the glucose produced is free to diffuse into the surrounding medium and be used by other cells. Some cells, called “cheaters”, do not express invertase but benefit from glucose produced by other cells [34]. Because cells incur a metabolic cost to produce invertase, cheaters have a reproductive advantage over invertase producers [34]. Thus, one might expect the population to eventually converge to a homogeneous population of cheaters. But, of course, a population composed solely of cheaters would soon starve. Conversely, if all cells produced invertase there would be a glut of glucose and less reproduction. There is a clear analogy between the yeast and farmers. The town maximizes its income when there is an optimal diversity of farmers so there are enough farmers planting tulips to meet the market demand but not so much as to glut the market. The town is penalized when too many farmers plant tulips because fewer farmers are left to plant marketable beans. Likewise, there is no need for all yeast cells expend energy making invertase and produce a glut of glucose; the “cheaters” can devote their energy to reproduction. If we define the population benefit function as the growth rate, there would be an optimal mix of slow-growing invertase producers and fast-growing “cheaters” that maximizes the growth rate of the colony as a whole. D.S. Wilson [26] writes that differences between individuals within a population may be “end product of natural selection” rather than “the raw material on which natural selection acts.” In other words, evolution may be selecting for organisms that maintain an optimal level of phenotypic variability. Genetic noise in gene translation or transcription [35–37] may also serve to maintain this level of variability.

The mathematical framework we developed here provides a tool for answering the question “What is the optimal level of diversity in a population that maximizes benefit?” We hope our work prompts new ways of thinking about and analyzing the effect of variability on a population.

## Supporting Information

S1 FileMathematical proof for convergence Sloppy Algorithm, convergence demonstrations, and differences between the Sloppy Algorithm and dithering.This document has five sections. Section **1** has a proof for the convergence of the Sloppy algorithm. Section **2** shows how the magic number is explicitly computed when ϕ∈N and *f*(*L*) = *L*. Section **3** gives the condition when a useful magic number can be found. Section **4** gives an example where a person is part of the Sloppy Algorithm and has the task of determining the absolute gray-scale value. Section **5** describes the differences between sloppy rulers and dithering.(PDF)
