# Supplementary material for: Optimizing Population Variability to Maximize Benefit
Source: PLoS One. 2015 Dec 9;10(12):e0143475. doi: 10.1371/journal.pone.0143475 (PMC4674128; doi:10.1371/journal.pone.0143475)
Supplement: S1 File — This document has five sections. Section 1 has a proof for the convergence of the Sloppy algorithm. Section 2 shows how the magic number is explicitly computed when ϕ∈N and f(L) = L. Section 3 gives the condition when a useful magic number can be found. Section 4 gives an example where a person is part of the Sloppy Algorithm and has the task of determining the absolute gray-scale value. Section 5 describes the differences between sloppy rulers and dithering. (PDF) [file pone.0143475.s001.pdf]

Supporting Information: File S1
Optimizing Population Variability to Maximize Benefit

Leighton T. Izu, Tamás Bányász, and Ye Chen-Izu

1 Convergence proof for Sloppy Algorithm

Notation and assumptions

Our goals in these appendices are:

1. Prove that the Sloppy Algorithm converges monotonically everywhere when ϕ is normally distributed and $f(L) = L$.
2. We will adapt the proof to the case where $f(L)$ is sigmoidal and ϕ is lognormal.
3. We will show how the magic number is derived when ϕ is normal and $f(L) = L$.
4. We show what it means to have a useful magic number.

Notation: (1) Strictly monotonically increasing, SMI. (2) Monotone convergence theorem, MCT. (3) $\mathcal{N}(\mu, s)$ is the normal distribution with mean μ and standard deviation s. (4) $\mathcal{LN}(m, s)$ is the lognormal distribution with median m and shape factor s.

Assumptions 1 and 2 hold throughout these appendices.

Assumption 1: Let $f(L)$ be strictly monotonically increasing (SMI) on $L = [0, 1]$ and let it be bounded by $0 \leqslant f(L) \leqslant 1$.

Assumption 2: Let $\phi(x, s)$ be a probability distribution function (pdf) and let $Q(L, s)$ be

$$\begin{aligned} Q(L,s) &= \int_{\Gamma(L)} \phi(t,s)\, dt && \text{(S1)} \\ &= \frac{1}{2}\left[1 + q(L,s)\right]. && \text{(S2)} \end{aligned}$$

The following rules define the Sloppy Algorithm.

1. Choose s_0 arbitrarily.
2. Compute $Q(L, s_0) = f_1$.
3. For any $i \geqslant 1$

$$\text{choose } s_i \text{ such that } Q(\lambda_i, s_i) = f_i \text{ where } \lambda_i \text{ solves } f(\lambda_i) = f_i. \tag{S3}$$

4. Compute the next f_{i+1} using

$$Q(L, s_i) = f_{i+1}. \tag{S4}$$

Remarks. Monotonicity of $f(L)$ is needed for the inverse function $f^{-1}(x)$ to exist. $Q(L, s)$ is monotonic in L because Q is the integral of a pdf, which is everywhere non-negative.

General lemmas

Lemma 1 (Dominance)**.** *If* $f_i < f(L)$ *then* $f_{i+1} > f_i$.

Proof: The proof hinges on the monotonicity of $f(L)$ and $Q(L, s)$. The monotonicity of $f(L)$ tells us that

$$\text{If } f_i \equiv f(\lambda_i) < f(L) \text{ then } \lambda_i = f^{-1}(f_i) < L. \tag{S5}$$

From (S3) we get $f_i = Q(\lambda_i, s_i)$ and from (S4) we get $f_{i+1} = Q(L, s_i)$. Suppose to the contrary that $f_{i+1} \leqslant f_i$ then $f_i = Q(\lambda_i, s_i) \geqslant Q(L, s_i) = f_{i+1}$. Because Q is monotonic in L if follows that $\lambda_i \geqslant L$. However, because of the monotonicity of $f(L)$ it follows [statement (S5)] that $f(\lambda_i) = f_i \geqslant f(L)$, which contradicts our assumption. Therefore, $f_{i+1} > f_i$ as claimed.

□

Lemma 1′. *If* $f_i > f(L)$ *then* $f_{i+1} < f_i$.

Proof is similar to Lemma 1.

Lemma 2 (Uniqueness)**.** $f(L)$ *is the unique accumulation point.*

Proof: Suppose there is $f(\tilde{L})$ which is the limit of the sequence of the Sloppy Algorithm and we assume without loss of generality that $f(\tilde{L}) < f(L)$. From (S4) we have

$$\lim_{i\to\infty} Q(L, s_i) = \lim f_{i+1} = f(\tilde{L}).$$

And from (S3) we have

$$\lim_{i\to\infty} Q\left(f^{-1}(f_i), s_i\right) = \lim f_i = f(\tilde{L}).$$

Equating these two expressions give

$$Q(L, s) = Q\left(f^{-1}(f(\tilde{L})), s\right).$$

But if $f(\tilde{L}) < f(L)$ then by statement S5 if follows that

$$f^{-1}(f(\tilde{L})) = \tilde{L} < f^{-1}(f(L)) = L.$$

But because Q is monotonic in L then $Q(L, s) > Q\left(f^{-1}(f(\tilde{L}), s\right)$ contradicting the equality. Therefore, $f(\tilde{L}) \geqslant f(L)$.

We can argue similarly that $f(\tilde{L}) \leqslant f(L)$ therefore, $f(\tilde{L}) = f(L)$ and thus proving uniqueness.

□

Remark: We proved Lemmas 1 and 2 without specifying f or ϕ so they hold generally.

Convergence of the Sloppy Algorithm when $\phi = \mathcal{N}$ and $f(L) = L$

The Sloppy Algorithm will not, in general, converge monotonically for any pair $\left(f(L), \phi\right)$. In the case where $f(L) = L$ and ϕ is the normal distribution, the Sloppy Algorithm converges monotonically everywhere. The proof given below can be adapted to study convergence of the Sloppy Algorithm for any $\left(f(L), \phi\right)$ pair.

Theorem 1 (Convergence Theorem)**.** *Let $f(L) = L$ on $L \in [0, 1]$. Let ϕ be the normal distribution $\mathcal{N}(L, 0, s)$ with mean of zero and standard deviation s. $Q(L, s)$ is defined by (S2) where Γ is the range from $1/2 - L$ to ∞. Then $\{f_i\}$ defined by recursion rules (S3) and (S4) converges monotonically to $f(L)$ everywhere.*

Note that in this case $f_i = \lambda_i$.

Proof: The key to the proof and understanding whether the sequence $\{f_i\}$ converges monotonically depends on the shape of the $s^*(L)$ curve. $s^*(L)$ solves the fixed point problem

$$f(L) = Q(L, s^*(L)). \tag{S6}$$

For the pair $\big(f(L), \phi\big) = \big(L, \mathcal{N}(L,s)\big)$, $Q(L,s)$ is

$$Q(L,s) = \frac{1}{s\sqrt{2\pi}} \int_{1/2-L}^{\infty} \mathrm{e}^{-z^2/2s^2} dz \tag{S7}$$

$$= \frac{1}{2}\left[1 + \operatorname{erf}\left(\frac{\sqrt{2}(2L-1)}{4s}\right)\right]. \tag{S8}$$

$s^*(L)$ is found by solving (S6) with $f(L) = L$,

$$s^*(L) = \frac{\sqrt{2}(2L-1)}{4\operatorname{erf}^{-1}(2L-1)}, \tag{S9}$$

which is shown in Figure A. Note that as $L \to 1/2$, $\operatorname{erf}^{-1}(2L-1) \to \frac{\sqrt{\pi}}{2}(2L-1)$ so $s^*(1/2) = 1/\sqrt{2\pi} \approx 0.4$. We now have the pieces needed to complete the proof.

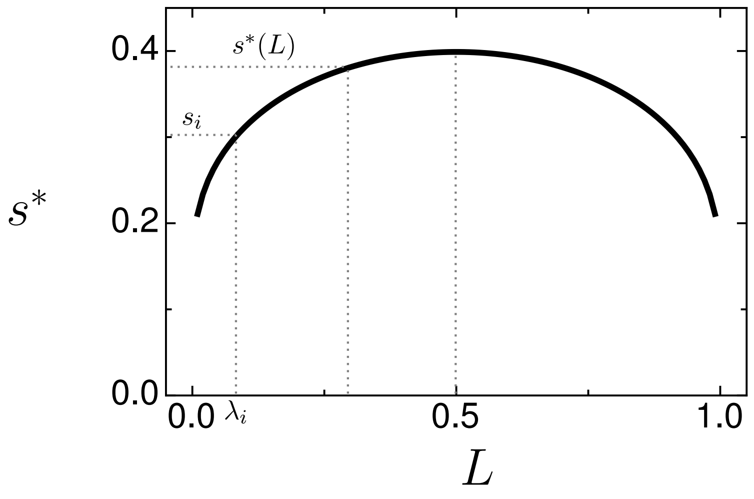

Figure A: **Optimal standard deviation s when ϕ is normally distributed and $f(L) = L$.**

Case 1A: Suppose λ_i and L lie on the same side of 1/2, say, $\lambda_i < L < 1/2$ (Figure A). From Lemma 1 we know that $\lambda_{i+1} > \lambda_i$ but we do not yet know

whether λ_{i+1} is greater than or less than L. Define z as

$$z(s) = \frac{\sqrt{2}(2L-1)}{4s}. \tag{S10}$$

Recall that $\lambda_{i+1} = Q(L, s_i) = Q(z_i)$ (step (S4) in the Sloppy Algorithm). Because $s_i < s^*(L)$ (see Figure A) and because $L < 1/2$ (which makes the numerator < 0) it follows that $z_i < z^*(L)$. Because Q is SMI in z, it follows that $\lambda_{i+1} = Q(z_i) < Q(z^*(L)) = L$. Thus $\{\lambda_i\}$ is a SMI sequence bounded above by L so by the monotone convergence theorem (MCT), the sequence converges to some $\tilde{\lambda}$. However, Lemma 2 tells us that $\tilde{\lambda} = L$.

Case 1A′: The cases where $L < \lambda_i < 1/2$, $1/2 < L < \lambda_i$, and $1/2 < \lambda_i < L$ can be handled in the same way to show $\{\lambda_i\}$ is a SM increasing (decreasing) bounded above (below) by L.

Case 1B: Suppose $\lambda_i < 1/2 < L$. We want to show that there is some $k > i$ for which $\lambda_k > 1/2$. Suppose no such k exists so for all $k > i$, $\lambda_k \leqslant 1/2$. From Lemma 1 we know that $\lambda_{i+1} > \lambda_i$. Therefore $\{\lambda_k\}$ is a SMI sequence bounded by $1/2$ so by the MCT $\lambda_k \to \tilde{\lambda}$. However, Lemma 2 demands that $\tilde{\lambda} = L$ therefore, contrary to our assumption, there must have been some k where $\lambda_k > 1/2$. Beyond this k, the situation is identical to Case 1A or 1A′.

Because i was arbitrary, it follows that any sequence $\{\lambda_i\}$ generated by the Sloppy Algorithm converges monotonically to L.

□

Figure B shows the monotonic convergence of the Sloppy Algorithm. Each colored path represents a different L. Note that for $L > 1/2$, $\lambda_{i+1} > \lambda_i$ while the opposite is true for $L < 1/2$.

The Sloppy Algorithm when $\phi = \mathcal{LN}$ and $f(L)$ is sigmoidal

Let

$$f(L) = \frac{L^n}{K^n + L^n} \text{ where } 0 \leqslant L \leqslant 1. \tag{S11}$$

Substituting $\phi = \mathcal{LN}$ into (S2) and integrating between 0 and ℓ gives

$$Q(\ell, m, s) = \frac{1}{2}\left[1 + \operatorname{erf}\left(\frac{\ln(\ell) - m}{\sqrt{2}s}\right)\right].$$

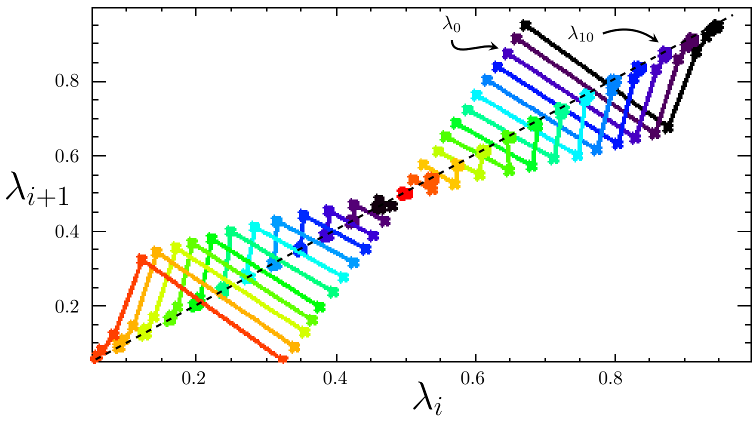

Figure B: **Global monotonic convergence when ϕ is normal and $f(L) = L$.** The $i+1$-st estimate of λ is plotted against the i-th estimate. Curved arrows point to λ_0 and λ_{10} for $L = 0.875$. Note that when $L > 1/2$, $\lambda_{i+1} > \lambda_i$ for all i, which indicates monotonic convergence. For $L < 1/2$, the opposite holds. In all cases, the initial value of s was 1.

$Q(\ell, m, s) = f(\ell)$ is satisfied when $s(\ell)$ is given by

$$s(\ell, m) = \frac{\ln(\ell) - m}{\sqrt{2}\text{erf}^{-1}\left(2f(\ell) - 1\right)}. \tag{S12}$$

Let $\bar{\ell}$ solve

$$2f(\bar{\ell}) - 1 = 0. \tag{S13}$$

Then m must be

$$m = \ln\left(\bar{\ell}\right). \tag{S14}$$

The plot of $s(\ell)$ is shown in Figure C.

Case 2A: $\lambda_i < L < \bar{\ell}$. From Lemma 1, $f_i < f_{i+1}$ but we don't know if f_{i+1} is greater than or less than $f(L)$. By definition $f_{i+1} = Q(L, s_i)$ and $Q(L, s^*) = f(L)$. From Figure C we see that $s_i > s^*(L)$. Let

$$z_i = \frac{\ln(L/\bar{\ell})}{\sqrt{2}s_i}.$$

Because $L < \bar{\ell}$, the numerator is < 0 so $z_i > z^*$. Therefore, $f_i < f(L) < f_{i+1}$ and we do not get monotonic convergence.

Case 2B: $\bar{\ell} < L < \lambda_i$. By Lemma 5, $f_{i+1} < f_i$ but we don't know whether f_{i+1} is greater than or less than $f(L)$. From Figure C we see that $s^* < s_i$. Therefore, $z^* > z_i$ (now the numerator is > 0) therefore, $f_{i+1} = Q(L, s_i) <$

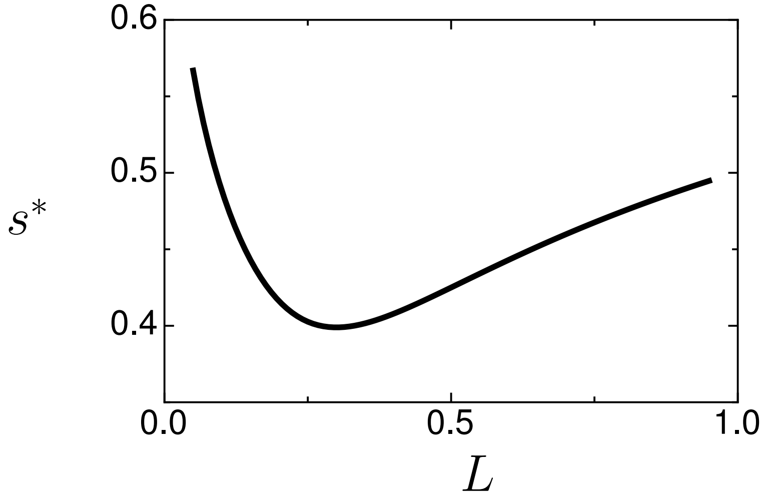

Figure C: **Optimal shape factor s when ϕ is lognormal and $f(L)$ is sigmoidal.** $s^*(L)$ is given by eqn. (S12)

$Q(L, s^*) = f(L)$. Thus, $f_i > f(L) > f_{i+1}$ and again we do not get monotonic convergence.

Figure D shows the nonmonotonic convergence for both $L < 1/2$ and $L > 1/2$.

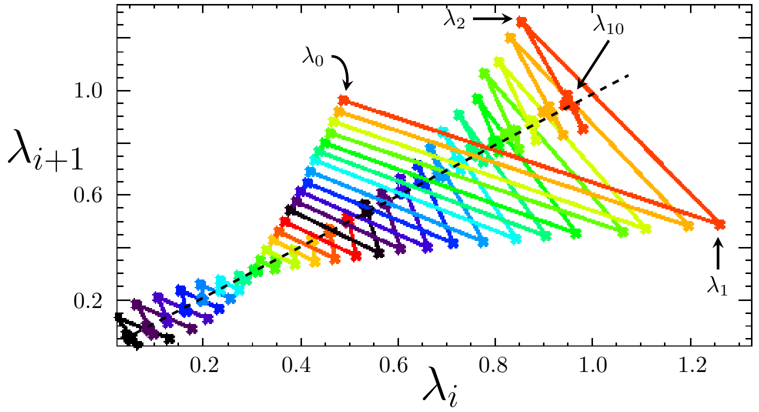

Figure D: **Nonmonotonic convergence of the Sloppy Algorithm when ϕ is lognormal and $f(L)$ is sigmoidal.** Plot is similar to Figure B. $f(L)$ is given by eqn. (S11) where $K = 0.3$. Although the Sloppy Algorithm converges, the convergence is nowhere monotonic.

Remarks: Using the same kinds of arguments we can show that for the cubic function $f(L) = L - (\gamma/2)L \cdot (L - 1/2) \cdot (L - 1)$ (for $\gamma \in [0, 4]$) and $\phi \in \mathcal{N}(0, s)$ the Sloppy Algorithm converges monotonically everywhere. However, when $\phi \in \mathcal{LN}(\ln(1/2), s)$ then the Sloppy Algorithm converges monotonically only for $L > 1/2$; convergence is oscillatory for $L < 1/2$.

2 Magic number for $\phi \in \mathcal{N}$ and $f(L) = L$

$s^*(L)$ solves the fixed point problem $Q(L, s^*) = L$. Writing $z = 2L - 1$ the fixed point problem becomes

$$\mathrm{erf}\left(\frac{\sqrt{2}z}{4s^*}\right) = z.$$

Because $L \in [0, 1]$ then $z \in [-1, 1]$. We use the approximation $\mathrm{erf}(x) \approx x$ for $x \in [-1, 1]$. Then the fixed point problem becomes

$$\frac{\sqrt{2}z}{4s^*} \approx \mathrm{erf}\left(\frac{\sqrt{2}z}{4s^*}\right) = z$$

from which it follows that

$$s^* \approx \frac{\sqrt{2}}{4} \equiv s_m.$$

Because this approximation holds over all $L \in [0, 1]$ it follows that s_m almost solves the fixed point problem for all L, that is, $Q(L, s_m) = L$.

3 Condition for having a useful magic number

When the benefit $B(L, \nu)$ is defined as (eqns. (9) and (11) in the main text)

$$\begin{aligned} B(L,\nu) \equiv \frac{\tilde{B}(L,\nu)}{D_m} &= \left[H(\nu)\nu - H(\nu-1)(\nu-1)\right] L b_1 \\ &+ \frac{b_2}{\beta}(1 - L\nu) - L|1-\nu| b_3 \end{aligned} \quad \text{(S15)}$$

or

$$B(L,\nu) = \frac{f(L)}{L}\nu - \frac{\nu^2}{2}, \quad \text{(S16)}$$

then $B(L, \nu)$ is maximized when $\nu = f(L)/L$. $s^*(L)$ solves the fixed point problem $Q(L, s^*(L)) = f(L)$ so B always is maximized when $s^*(L)$ is used. For arbitrary s, $Q(L, s) \neq f(L)$ so $\nu(s) = Q(L, s)$ will not maximize B. We'd like to replace the continuum $s^*(L)$ with a single magic number s_m that almost maximizes $B(L, \nu)$ for all L.

Clearly, the closer $Q(L, s_m)$ approximates $f(L)$ for *all* $L \in [0, 1]$, the closer $B(L, \nu)$ will be it to its maximum value. In other words, $Q(L, s_m)$ should "look

like" $f(L)$ in the sense that $Q(L, s_m)$ is close to $f(L)$ everywhere. The natural metric for this is the maximum norm,

$$d\big(Q(L, s_m), f(L)\big) = \max\left(\big|Q(L, s_m) - f(L)\big| \text{ for all } L \in [0, 1]\right). \tag{S17}$$

If $d\big(Q(L, s_m), f(L)\big)$ is small then $B(L, \nu(s_m)) \approx B(L, \nu(s^*))$, meaning the benefit is nearly maximized.

The reason the normal distribution gave such poor performance when $f(L)$ was sigmoidal and the lognormal distribution gave excellent performance is because $Q_{\mathcal{N}}(L, s_m)$ (the integral in (S2) when $\phi \in \mathcal{N}$) does not look sigmoidal whereas $Q_{\mathcal{LN}}(L, s_m)$ ($\phi \in \mathcal{LN}$) looks remarkably sigmoidal.

4 Determining the grayscale level using the Sloppy Algorithm

Algorithms that work well in a computer simulation can fail miserably outside of a simulation. We tested whether the Sloppy Algorithm would work when a human was part of the iteration loop. The problem was to see if the Sloppy Algorithm could be used by a person to determine the absolute, as opposed to a relative, magnitude of a quantity. Examples of this kind of task is determining the brightness of a variable star by eye (http://www.aavso.org/) or the weight of an ox [1].

The specific problem task was to determine the gray scale value of an image. The image, a square displayed on the computer monitor, had a gray scale value between 0 and 255. Next to the test square, a comparison square of equal size whose gray level was randomly chosen from a normal distribution with a mean level of 128 and standard deviation s_i was shown. The person had to decide whether the test square was brighter or dimmer than the comparison square. After making N comparisons, $\bar{\lambda}_i$ was calculated from $\bar{\lambda}_i = n/N$, where n was the number of times that a test square was judged brighter than the comparison square and i is the iteration number. This is step [4] (eqn. (S4)) in the Sloppy Algorithm. Based on $\bar{\lambda}_i$ a new s_i that solved eqn. (S3) was determined. Fig. E shows results from three tests (from 2 subjects). The dashed lines mark the correct gray level L. The initial s value was set to $1 \times \Delta$ (filled circle) or $0.1 \times \Delta$ (filled square) where $\Delta = 256$ equals the range of possible gray values. These initial s values were chosen so that the first estimate $\bar{\lambda}_1$ would be far from L (the program "knew" the value of L but the person did not) thereby

allowing us to see how the estimates converged to L. The convergence to the correct grayscale value is similar to that seen in Figure 2 in the main text except that the convergence is nonmonotonic. Nonmonotonicity arises from the finite number of decisions (N) that were made; simulations show that the convergence becomes monotonic as $N \to \infty$. We used $N = 150$ to get good estimates of $\bar{\lambda}_i$ but making such a large number of decisions (150×6 iterations = 900 decisions) is tiring. Therefore, we tested whether setting s_0 to the magic number would hasten the convergence. The results (open triangle) in this case show that even on the first iteration the estimate (71.4) is already close to the correct value (70).

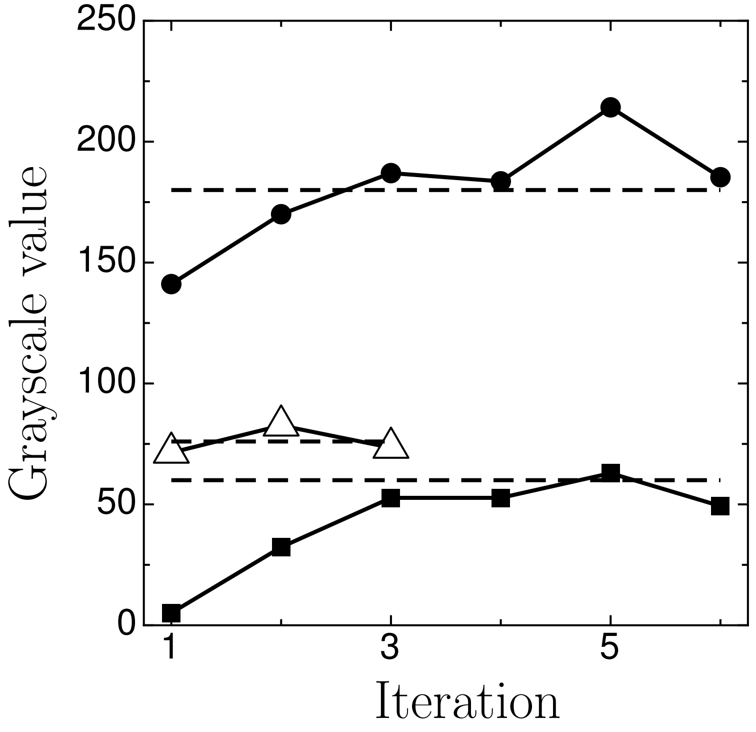

Figure E: **Evolution of grayscale estimation using Sloppy Algorithm.** Dashed lines indicate correct grayscale values. Subject 1, squares and triangles; subject 2, circles. Convergence is immediate when initial s equalled the magic number (triangles).

For this example one observer makes N decisions while in the main text each of the N rulers makes one decision. These two approaches are mathematically equivalent.

5 Sloppy Algorithm and sloppy rulers versus dithering

Sloppy rulers when combined with the Sloppy Algorithm can make accurate, high-resolution measurements even though each sloppy ruler has the lowest possible resolution. Dithering is a technique that can also improve measurement resolution [2] and has long been used to reduce quantization errors of analog-to-digital conversion [3]. Noise is essential in both dithering and sloppy rulers.

However, sloppy rulers and dithering are different mathematically and in their arenas of application. In dithering the output signal is the average of both positive and negative excursions over many quantized states (256 states in an 8-bit analog-to-digital converter) centered around the input signal. By contrast, sloppy rulers average over only two states, zero and one.

This difference in what quantities are averaged is important in determining what the optimal noise level, s, should be to get accurate measurements. For dithering any s larger than half of the quantization step size will produce an accurate output [2]. For sloppy rulers, there is a unique s for each input value L that produces an accurate output, which the Sloppy Algorithm finds. Choosing s arbitrarily produces estimates of L shown in Fig. 3A in the main text. The y-axis is the estimate of L; only by happenstance does the estimate match the true value of L.

The Sloppy Algorithm and dithering are useful in different systems. Sloppy rulers represent a wide class of systems that make binary decisions. Such systems include yes-or-no voting in politics, all-or-none protein expression in cells, choice of crops to plant. Dithering, on the other hand, is useful when there are many signal levels as in analog-to-digital converters and in smoothing out pixelation in images.

References

[1] Galton F. Vox Populi. Nature. 1907;75:450–451.

[2] Etchenique R, Aliaga J. Resolution enhancement by dithering. Am J Physics. 2004;72:159–163.

[3] Lipshitz SP, Wannamaker RA, Vanderkooy J. Quantization and dither: A theoretical survey. J Audio Eng Soc. 1992;40:355–375.
